# The Impact of a Motor Imagery-Based Training Program on Agility, Speed, and Reaction Time in a Sample of Young Tennis Athletes during Ramadan Fasting: Insights and Implications from a Randomized, Controlled Experimental Trial

**DOI:** 10.3390/nu12113306

**Published:** 2020-10-29

**Authors:** Sofien Fekih, Mohamed Sami Zguira, Abdessalem Koubaa, Imed Ghariani, Hamdi Zguira, Nicola Luigi Bragazzi, Mohamed Jarraya

**Affiliations:** 1 Higher Institute of Sport and Physical Education of Gafsa, Gafsa 2100, Tunisia; fkih2007@hotmail.com (S.F.); sami-zguira@hotmail.fr (M.S.Z.); abdessalemkoubaa@gmail.com (A.K.); hamdi.zguira@gmail.com (H.Z.); 2 Institute of Sport and Physical Education of Ksar Said, University of Manouba, Manouba 2010, Tunisia; 3 Research Laboratory: Education, Motricity, Sports and Health (EM2S, LR19JS01), Higher Institute of Sport and Physical Education of Sfax, University of Sfax, Sfax 3100, Tunisia; jarrayam@yahoo.fr; 4 Department of Physiology and Lung Function Testing, Faculty of Medicine Ibn-El-Jazzar, University of Sousse, Sousse 4000, Tunisia; 5 Laboratory of Pharmacology, Faculty of Medicine of Sfax, University of Sfax, Sfax 3100, Tunisia; 6 National Centre of Medicine and Science in Sport, Tunis 2000, Tunisia; imedghariani2@gmail.com; 7 Postgraduate School of Public Health, Department of Health Sciences (DISSAL), Genoa University, 16132 Genoa, Italy; 8 Department of Neuroscience, Rehabilitation, Ophthalmology, Genetics, Maternal and Child Health (DINOGMI), Section of Psychiatry, Genoa University, 16132 Genoa, Italy; 9 Laboratory for Industrial and Applied Mathematics (LIAM), Department of Mathematics and Statistics, York University, Toronto, ON M3J 1P3, Canada; 10 Higher Institute of Sport and Physical Education of Sfax, University of Sfax, Sfax 3100, Tunisia

**Keywords:** Ramadan fasting, performance, agility, speed, reaction time, motor imagery-based training program, sport psychology

## Abstract

The objective of this study was to explore whether a training program incorporating motor imagery could have an effect on physical performance outcomes in terms of agility, speed, and reaction time in a sample of tennis athletes who fasted during the month of Ramadan. Recruited subjects were 27 young male tennis players, randomly allocated to two groups: the imaging training group (*n* = 13) and a control group (*n* = 14). The study was designed as a randomized, controlled experimental study. The control group was engaged in watching videos concerning the history of the Olympic Games, whereas the motor imagery group followed a motor imagery-based training program. Physical performance outcomes were assessed during four sessions (one before Ramadan and three during Ramadan) by means of field tests. Our results revealed a drop in all performance outcomes measured in the middle and at the end of Ramadan for both groups (*p* < 0.01). The effect of the group × time interaction (*p* < 0.01) was reported for all physical performance outcomes measured for the two groups. This drop in performance was greater for the control group compared to the motor imagery group in the middle and at the end of Ramadan. This study showed that fasting during Ramadan reduced agility, speed, and reaction time performance for tennis players. A motor imagery-based training program could be an effective approach to reduce the effect of fasting during Ramadan and stabilize physical performance outcomes in terms of agility, speed, and reaction time for male tennis players.

## 1. Introduction

The religious tradition of Islam is based on five pillars, one of which is represented by Ramadan fasting, during which Muslim individuals are asked to refrain from consuming food and beverages as well as from sexual intercourse during the day. This fasting period extends over a month and can reach 17–18 h per day, depending on the geographic location, which affects daylight hours and, therefore, fasting duration. For example, in recent years Ramadan has occurred during the summer, coinciding with several national or international sporting events (like the national tennis championship in Tunisia or the Roland-Garros tennis tournament). As a result, Ramadan fasting can cause sleep-wake disturbances as well as changes in dietary uptake, which can affect physical performance in sports [1]. For this reason, scientists, nutrition practitioners, and researchers in the field of sport should find effective training strategies to help Muslim tennis players deal with Ramadan fasting as it can negatively impact their sports performance [2]. The review of the scientific literature, regarding the impact of fasting during Ramadan on sports performance, reveals several findings which confirm the decline in physical performance [3,4,5], due to the inability to preserve the same training load with respect to the before-Ramadan period and the proposed training method.

Performance in tennis is the result of a combination of technical, tactical, and physical variables and psychological determinants. In this way, physical performance is described as an interaction between speed, power, agility, and aerobic qualities [6,7,8]. For this reason, tennis is classified as an intermittent high-intensity sport [9]. Indeed, the analysis of a tennis match shows that changes of direction, movements, and reaction time to move require the integration and coordination of different skills. The notion of agility is necessary for the movements implemented by tennis players [10]. Therefore, Ramadan fasting could be detrimental for the outcomes of tennis players, due to its potential metabolic effects. Although specific and continuous physical and technical training can improve the physical performance outcomes of tennis under normal conditions [10,11], in special situations like Ramadan, it may not be enough to help counter or lessen the negative effects of fasting, because the strategies that athletes can adopt to prevent a decline in performance during Ramadan are limited given the restriction of food and water intake imposed during the day. It would, as such, be imperative to resort to alternative strategies emphasizing nutritional aspects. Therefore, in order to manage the effects of Ramadan fasting, sports coaches should find effective approaches to stabilize or improve the physical performance outcomes of fasting Muslim athletes.

Different studies have shown that incorporating a motor imagery training program into the regular training programs of athletes could help optimize the physical performance of various sports and physical activities requiring strength [12]. Indeed, many trainers and mental coaches use motor imagery-based programs during training to enhance athletes’ physical performance outcomes [13,14,15].

However, there is a dearth of information concerning the impact of training strategies incorporating motor imagery modules on the physical performance outcomes in tennis during the fasting of Ramadan. It is of paramount importance to assess the potential benefits of maintaining performance levels through imagery, given the metabolic alterations and performance limitations imposed as a result of Ramadan. Therefore, this trial was an attempt to investigate the effect of a motor imagery-based training program on physical performance outcomes, by performing a randomized controlled study with tennis athletes who fast during the month of Ramadan.

Two working hypotheses have been formulated. First, we speculated that Ramadan fasting has a negative effect on physical tennis performance (in terms of agility, speed, and reaction time). As second hypothesis, we hypothesized that training programs incorporating motor imagery could curb or decrease the detrimental impact of Ramadan fasting on tennis-related outcomes (in terms of agility, speed, and reaction time).

## 2. Material and Methods

The protocol of the present study has been in-depth reviewed and approved by the “Ethics Committee for the Protection of Southern People” (C.P.P.SUD), Sfax, Tunisia: protocol reference C.P.P.SUD N ° 0057/2016. The present study, a four-week randomized controlled experimental study conducted during the month of Ramadan in 2016, was carried out based on the latest version of the Helsinki Declaration and its subsequent amendments. The registration code for the trial is PACTR202006847771700.

### 2.1. Sample Size Computation

The null hypothesis (*H*_0_) was formulated as *m*_1_ = *m*_2_, whereas the alternative hypothesis (*H_a_*) was formulated as *m*_1_ = *m*_2_ + *d*, with *d* being the difference between the two means. *n*_1_ is the sample size required for the experimental group, and *n*_2_ is the sample size requested for the control group, with *N* being the total sample size requested. *N* was computed by means of the following Equation (1) [16]:

(1) $$N=\frac{\left(r+1\right)\cdot {\left(Z_{\frac{\alpha }{2}}+Z_{1-\beta }\right)}^{2}\cdot \sigma^{2}}{r\cdot d^{2}},$$

where *Z_α_* is the normal deviate achieving statistical significance = 1.67 (5% level of significance), *Z*_1−*β*_ is the normal deviate at 1 − *β*% power with *β*% of type II error (0.82 at 80% statistical power), and *r* is calculated as the *n*_1_/*n*_2_ ratio (*r* = 0.69 gives the sample size distribution as 1:1.5 for two groups). *σ* and *d* are the pooled standard deviation (SD). These values were computed based on a similar hypothesis formulated in similar settings [17].

### 2.2. Participants

Twenty-seven tennis players voluntarily participated in this study, training regularly in clubs for 2 h a day, usually three times a week. Given the inclusion criteria utilized in other surveys with racket sports athletes [13,18], eligible subjects should: (1) be a tennis athlete for at least 2 years, (2) train in tennis at least 6 h a week, and (3) enter the national tennis championship.

Moreover, they should be willing to fast during the day (from early dawn to sunset) for the entire period of Ramadan. These players were divided into two groups, a control group of 14 players and an experimental mental imagery group of 13 players. We checked that there was no difference in groups by demographic parameters (sex and age), anthropometric parameters (height and body mass), and years of experience. These participants have experience in mental imagery-based training. All participants provided a written, informed agreement before data collection. Before the experiment, subjects in the experimental group had filled in the French version of the “Movement Imaging Questionnaire-Revised Second version” (MIQ-RS) [19]. The anthropometric data of the experimental and control groups and the scores of the questionnaire for the experimental group are presented in Table 1.

### 2.3. Procedure

This is a 4-week, randomized controlled experimental survey carried out during Ramadan of 2016. The two groups (imagery training group and control group) of young male tennis athletes completed the same physical/technical training plan over Ramadan, 2 h per afternoon training session from 5 p.m. to 7 p.m.

The control group watched videos regarding the Olympic Games, whereas the imagery training group was engaged in motor imagery training. Three weekly motor imagery training sessions were performed, spread over a 48-h period, for a total of 12 sessions over a 4-week period. The sessions were held at 30-min intervals between the end of the physical/technical training session and the start of the motor imagery training session, which is in agreement with Murphy [20]. All the motor image training sessions lasted approximately 15 min in a calm environment (near the tennis court), where the athletes wore the clothes that they wear usually during competitions [21]. Before each motor imagery training session, videos of tennis players performing technical gestures were used to facilitate the imaginative ability of the athletes allocated to the experimental group, in agreement with Battaglia [14]. In the present study, the mental training program was inspired by the work of Li-Wei [22], to justify the association of imagery with physical practice, and of Meacci [23], to legitimize the use of body simulations of movement. Each session was distributed as follows:

- First step: the imaging modality envisaged was external. This stage was managed by a video recording, which includes sequences for rapid movements to the next striking point, changes of direction in different axes, fixings for ground support, then rapid engagement to get in motion, powerful, and precise services. Participants were asked to mentally see themselves as if they were watching each other on a video screen. The steps were: (A) to think of a situation in the first person, (B) to imagine moving quickly to the next striking point, (C) to imagine changing direction in different axes, (D) to imagine fixing solid supports to the ground and then quickly leaving these supports to start in motion, and (E) to imagine performing powerful and precise services, by taking an interval of about 10 s between each imagination act.
- Second step: the imaging modality considered was internal. The participants were invited to experiment, and feel the sensations that were evoked in a real situation of physical practice, for the different skills already seen in the video sequences, by taking an interval of about 10 s between each imagination. During these evocations, the participants, who felt the need, could speak softly or mimic the movement. The participant used here the technique of body simulation of movement which has proven to be the most effective [23].
- Third step: Through informal discussions, the experimenter explains to participants the usefulness and effects of imagery. He stresses the importance of the adequacy between the goal pursued (building confidence or improving the technical and physical quality of the gesture) and the modality or perspective of imagery used. Chronometers of the type digital stopwatch (iSport JG021 Pro) were equipped for the athletes in order to properly control the duration of the mental simulation of the 10 imagination acts of each session.

Two coaches, experts in motor imagery training, provided interventions in the experimental group. These coaches attended the 12 training sessions in motor imagery for the imagery training group or videos for the control group, with the purpose of avoiding any bias between the groups.

**Figure 1:**
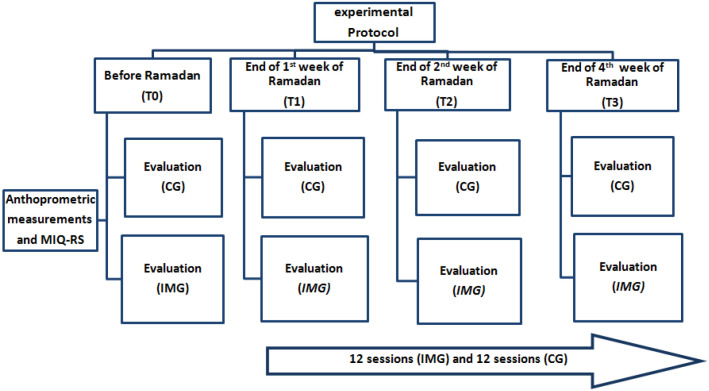
Experimental protocol adopted in the present study. Note. IMG = imagery training group; CG = control group; MIQ-RS = The Movement Imagery Questionnaire-Revised Second edition version.

The outcomes of the participants (agility, speed, and reaction time) were measured 48 h before, during, and at the end of the 4 weeks of the intervention during Ramadan fasting, as pictorially represented in Figure 1.

### 2.4. Measures

Concerning the calorie intake, the assessment of food consumption was carried out by the participants over a period of 3 consecutive days using a food consumption diary. The nutritional analysis was carried out by a nutritionist based on the Tunisian food composition table. The average daily calorie intake as well as the percentages of carbohydrates, fats, and proteins contained in consumed foods was calculated (Table 2). To evaluate the imaginative capacity of athletes, the MIQ-RS [19] was used.

For measuring agility, the MAT-Agility test is a modification of the *t*-test protocol. In this new version, the total distance traveled is 20 m. The procedure for this test is the same as for the *t*-test [24]. More specifically, this test assesses the speed of players while measuring their agility. Indeed, it offers 3 different racing styles (straight line, lateral movements, and backward racing) and, therefore, requires great qualities in terms of speed and agility, as found in tennis-related activities. Each player had 3 trials separated by 3 min of rest for complete recovery. We evaluated the time using photoelectric cells.

**Figure 2:**
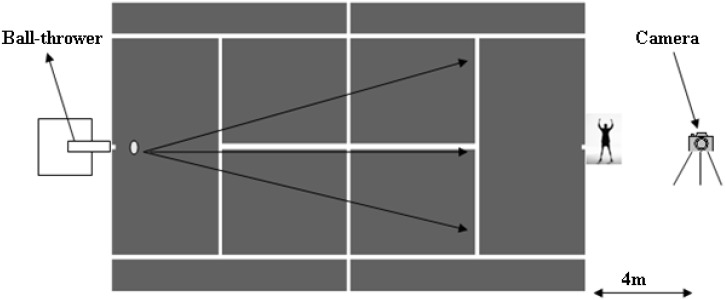
Reaction speed test following visual perception.

For the speed measurement, the ZIG-ZAG test was employed, which makes it possible to assess speed over short distances as well as the ability to change direction. We proposed a race over 3 m, a change of direction to the right over 2 m, a race of 4 m, a second change of direction to the left and a final race of 3 m, as proposed by the French tennis federation. Each player had 5 separate passages of 1:30 so that each passage was executed at maximum speed. In order to calculate the reaction time, the players were filmed using a digital video camera type (JVC GC-PX10) at a resolution of 250 frames per second. This was placed behind the receiver and was used to record when the ball was thrown and the first reaction of the raisers. This method was inspired by the work of Avilés [25]. Before performing the tests, each player was familiarized with the nature of the experimental task and the corresponding instructions, such as being placed in the receiving position, behind the baseline of the field. A ball thrower type Universal Sport Playmate Portable was placed on the baseline on the other side of the field. This ball launcher was programmed to make 10 throws at a constant speed of 100 km/h, at different places on the ground, with the same type of throw (service), so that the participant knew neither the time nor the type of the next launch. A total of 10 attempts were recorded for each player and the frame-by-frame analysis was performed every 4 ms (Figure 2). A Dell laptop with Quick Time 7 Player software was used, with an auxiliary screen. Measurements of height and body mass were made using a board and electronic scale (Tanita, Tokyo, Japan), respectively.

To examine our hypotheses regarding the effects of motor imagery training on physical performance outcomes measured in tennis athletes, during Ramadan fasting, factor analyses were performed. Means and standard deviations (SD) were used to describe all the variables (physical tennis performance, height, body mass, years of training, age, and MIQ-RS scores).

### 2.5. Statistical Analyses

Before proceeding with statistical analyses, the normality of the distributions was tested by means of the Shapiro–Wilk method. When this test was not significant (*p* > 0.05), the assumption of normality was not violated. An analysis of variance (ANOVA) with repeated factor measurements (Ramadan period (before/during/after) × motor imagery (with or without)) was performed on the variables of tennis physical performance and body mass values during the session before (T0), at the beginning (T1), in the middle (T2), and at the end (T3) of Ramadan fasting. Student’s *t*-test with Bonferroni correction was used to make pairwise comparisons.

## 3. Results

The basic descriptive data are reported in Table 1. No significant differences could be identified between the experimental and control groups before starting the intervention, indicating the homogeneity of the two groups. Table 2 shows the means (±SD) of the daily calorie intake and the percentages of carbohydrates, fats, and proteins contained in consumed foods, recorded during the two experimental periods (before Ramadan and during the fourth week of Ramadan). No significant differences could be identified for daily caloric intakes and percentages of carbohydrates, lipids, and proteins during the two periods.

Table 3 reports the anthropometric values of the two groups (imagery training group and control group) at different time points (namely, before, at the beginning, in the middle and at the end of Ramadan fasting). Significant differences could be observed for body mass and body mass index (BMI), in the middle (T2) and at the end of Ramadan (T3) compared to before Ramadan (T0) for the two groups.

Two-way ANOVA with repeated measurements of body mass revealed a significant interaction of Ramadan period (before/at the beginning/in the middle/at the end) × body mass (F (3.108) = 28.59; *p* = 0.001; *ηp*^2^ = 0.443). The fall in BMI was not recorded until the end of Ramadan. Statistical analysis showed a significant main effect (F (3.108) = 25.7; *p* = 0.001; *ηp*^2^ = 0.416).

**Figure 3:**
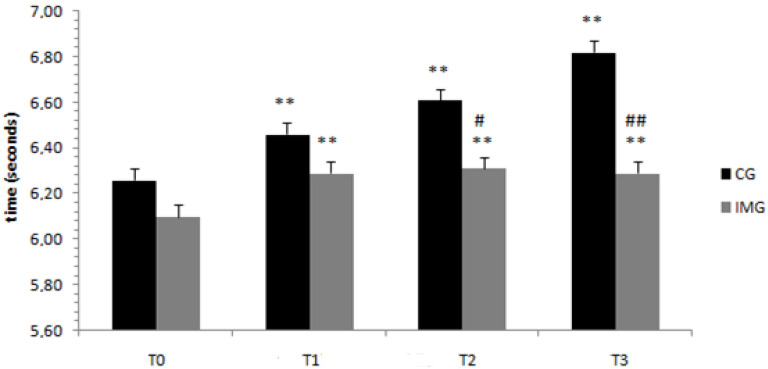
Means and standard deviations of the MAT-Agility test scores, before, at the beginning, in the middle, and at the end of Ramadan for the two groups (control group, CG, versus experimental group, imagery training group or IMG). CG = control group, IMG = imagery training group. ^##^, ^#^ Significantly different from the control group at *p* < 0.05 and *p* < 0.001, respectively. ** Significantly different from before Ramadan at *p* < 0.001.

Regarding the MAT-Agility test, the ANOVA results showed a significant interaction of Ramadan period (before/at the beginning/in the middle/at the end) × motor imagery (with or without) (F (3.108) = 16.69; *p* < 0.001; *ηp*^2^ = 0.317). Results revealed that in the motor imagery condition, the means of the scores of the MAT-Agility test were greater at the beginning (T1), in the middle (T2), and at the end of Ramadan (T3) compared to before Ramadan (T0) (*p* < 0.001). A similar trend could be observed for the training condition without motor imagery at *p* < 0.001 (Figure 3). With regard to the difference between the groups in the same periods, the means of the times of the MAT-Agility test were higher for the control group than for the imagery training group in the middle (T2) (*p* < 0.042) and at the end of Ramadan (T3) (*p* < 0.002).

**Figure 4:**
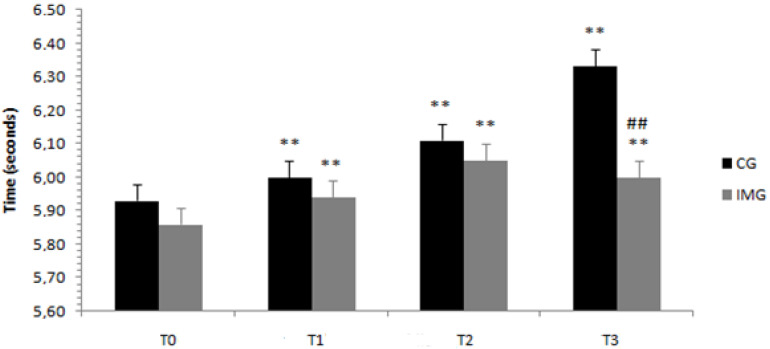
Means and standard deviations of the ZIG-ZAG test scores, before, at the beginning, in the middle, and at the end of Ramadan for the two groups (control group, CG, versus experimental group, imagery training group or IMG). CG = control group, IMG = imagery training group. ^##^ Significantly different from the control group at *p* < 0.001. ** Significantly different from before Ramadan at *p* < 0.001.

Regarding the ZIG-ZAG speed test, the ANOVA results showed a significant interaction of Ramadan period (before/at the beginning/in the middle/at the end) × motor imagery (with or without) (F (3.108) = 51.69; *p* < 0.001; *ηp*^2^ = 0.589). In the motor imagery condition, the ZIG-ZAG speed test time averages were greater than at the beginning (T1), in the middle (T2), and at the end of Ramadan (T3) with respect to before Ramadan (T0) at *p* < 0.003, *p* < 0.001, and *p* < 0.001, respectively. A similar trend could be observed for the training condition without motor imagery at *p* < 0.003, *p* < 0.001, and *p* < 0.001, respectively. With regard to the difference between the groups in the same periods, the ZIG-ZAG speed test time averages were higher for the control group than for the imagery training group at the end of Ramadan (T3) (*p* < 0.002), as shown in Figure 4.

**Figure 5:**
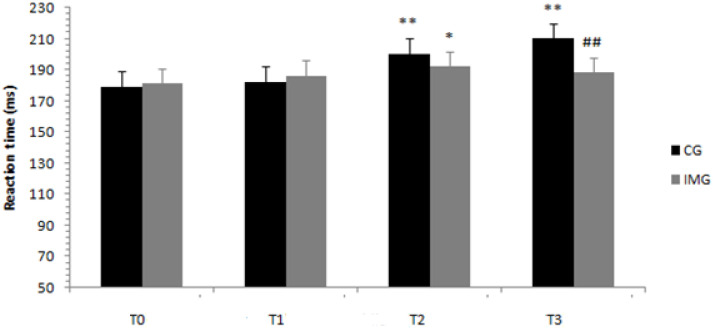
Means and standard deviations of the reaction time, before, at the beginning, in the middle, and at the end of Ramadan for the two groups (control group, CG, versus experimental group, imagery training group or IMG). CG = control group, IMG = imagery training group. ^##^ Significantly different from the control group at *p* < 0.001. **, * Significantly different from before Ramadan at *p* <0.05 and *p* < 0.001, respectively.

Regarding the reaction time, the ANOVA results showed a significant interaction of Ramadan period (before/at the beginning/in the middle/at the end) × motor imagery (with or without) (F (3.108) = 8.1; *p* < 0.001; *ηp*^2^ = 0.184). Results revealed that in the motor imagery condition, the mean reaction times were greater in the middle of Ramadan (T2) compared to before Ramadan (T0) (*p* < 0.014). For the training condition without motor imagery, the mean reaction times were greater in the middle of Ramadan (T2) and at the end of Ramadan (T3) compared to before Ramadan at *p* < 0.001 and *p* < 0.001, respectively. With regard to the difference between the groups in the same periods, the means of the reaction times were higher for the control group than for the imagery training group only at the end of Ramadan (T3) at *p* < 0.001, as shown in Figure 5.

## 4. Discussion

The aim of the present study was to examine whether motor imagery training for young male tennis athletes who partake in Ramadan fasting could have an effect on physical performance outcomes (namely, agility, speed, and reaction time of the receiver). Two hypotheses have been formulated. First, Ramadan fasting has a negative effect on short-term maximum physical performance outcomes for tennis athletes who fast. Second, the motor imagery training program, combined with physical/technical training, counteracts or, at least partially, mitigates the negative impact of Ramadan fasting on tennis athletes’ physical performance outcomes.

### 4.1. Effects of Ramadan Fasting on Physical Performance Outcomes of Tennis Athletes

Our results show a time increase as measured on the MAT-Agility test and speed test (ZIG-ZAG) for the control group. This decrease in performance has been observed by Memari [4] in young taekwondo players during the second and fourth weeks of Ramadan. In addition, Hammouda [26] observed that performance in the repeat sprint test was lower at the end of Ramadan in the afternoon compared to before Ramadan among 17-year-old football players. Contrary to our results, Chaouachi [27] noted no difference in time in 5, 10, and 30 m sprints during Ramadan compared to before Ramadan, in young 18-year-old judokas. It could be due to the nature of the tests, the participants in the tests, or the evaluation time. Regarding the reaction time, our results show an increase in the reaction time of the receiver in the middle (T2) and at the end of Ramadan (T3) compared to before Ramadan (T0). This result was also observed in young karateka [28]. Indeed, Bouhlel [28] showed an increase in reaction time at the end of Ramadan compared to before Ramadan under the condition of rest.

Other studies on sedentary subjects have also reported impaired alertness [29] and attention [30] during Ramadan. The reaction time was found to be extended from the end of the first week of the fast [31]. In contrast, Gutierrez [32] found no change in reaction time in trained subjects, fasting for 3 days. These different results may reflect differences in the nature of the fast (intermittent or continuous), the initial state of the subjects, the local environment (temperature and humidity), differences in the type of the exercise performed, and of the time of year when the fast was observed. This reduction in physical performance outcomes observed during the month of Ramadan is probably not due to fasting-induced dietary uptakes. Indeed, in agreement with previous studies [33,34], we observed no difference in caloric intake as well as the percentages of lipids, carbohydrates, and proteins contained in food, during Ramadan compared to before Ramadan.

The drop in performance in this month of fasting could be attributed to an alteration in the sleep–wake cycle. In this context, the total duration of sleep for our athletes would have been reduced given the time difference in food intake during Ramadan. Indeed, Waterhouse [35] reported that during Ramadan, Muslims continue to eat and drink until late hours at night, which is likely to prevent falling asleep. Then, they wake up for the last meal before dawn. These disturbances could reduce the duration of night sleep. The fatigue induced by this partial sleep deprivation could explain the decline in performance during Ramadan.

Moreover, Ramadan fasting is generally associated with a reduction in daily fluid intake, especially when fast takes place in summer. A warm environment combined with fasting can result in dehydration, which may decrease muscle performance [1] and be responsible for the decline in physical performance outcomes. In this context, Fekih [34] showed a reduction in body mass at the end of Ramadan compared to the day before Ramadan in the afternoon. This decrease could be due to a loss of body water. Likewise, Sweileh [36] observed severe dehydration associated with Ramadan fasting, with a significant reduction in body mass. The negative effects of hypohydration on sports performance are well documented in the literature [2]. Maughan [37] reported that 2% dehydration could cause a reduction in physical performance outcomes, with a 5% reduction potentially decreasing performance by up to 30%. We have observed significant differences in body mass at the end of the fasting period, which could explain the drop in performance outcomes reported in this study.

### 4.2. Effects of a Motor Imagery Training Program on Physical Performance Outcomes in Tennis during the Ramadan Fast

Results of this study revealed that the motor imagery-based training program during Ramadan reduces the negative effect of fasting on physical performance outcomes in tennis (agility, speed, and reaction time) confirming our hypotheses. This result is consistent with those findings showing an enhancement in the outcomes of physical tasks thanks to a training program based on motor imagery modules [14,17].

Concerning the times measured by means of the agility and speed tests, results of this study show, on the one hand, significant differences in the middle (T2) and at the end of Ramadan (T3) compared to before Ramadan (T0), for the two groups (imagery training group and control group). On the other hand, results show significant differences in favor of the imagery training group compared to the control group at the end of Ramadan (T3). This leads us to conclude that the effect of a training program in motor imagery did not show an improvement in the performance of agility and speed following its inhibition during Ramadan, but it limited the drop in performance measured during Ramadan and therefore, it has a beneficial effect on these physical performance outcomes, being able, at least partially, to counter the effects of Ramadan fasting. Several studies have shown an improvement in sports performance with the practice of motor imagery [38,39]. Concerning the reaction time measured in this study, results show, on the one hand, an increase in the reaction time just in the middle of Ramadan (T2) for the imagery training group, whereas for the control group, an increase in the reaction time was reported in the middle (T2) and at the end of Ramadan (T3), compared to before Ramadan at T0. On the other hand, significant differences in favor of the imagery training group compared to the control group could be found at the end of Ramadan (T3). As such, we can say that the motor imagery-based training program limited the increase in reaction time during Ramadan and, therefore, it has a beneficial effect in countering and slowing the effects of Ramadan fasting on physical performance outcomes. In this sense, no previous studies have been found that examine the effect of training with motor imagery on reaction time during Ramadan. On the other hand, the beneficial effects of motor imagery on physical performance outcomes in general have been highlighted in previous work as successful intervention strategy. For instance, the study by Davis [40] supports the idea that the use of motor imagery by athletes can stimulate/improve their personal achievements, by modifying psychological and physiological conditions, which is beneficial for athletic performance in general. Indeed, in our study we reported a delimitation of the negative effect of fasting on performance outcomes in terms of agility, speed, and reaction time for the imagery training group. Moreover, Fekih [34] found similar results on tennis service performance. From a psychological standpoint, approaches like imagery are applied repeatedly to improve physical performance across multiple emotional and motivational functions [41]. From a neuroscientific perspective, motor imagery is speculated to result in an increase in neuronal activities and neurophysiological processes in several executive areas of the brain, which would lead to an improvement in performance outcomes [40,42]. Moreover, at the muscle activation level, mental imagery training in the month of Ramadan can have a positive effect on performance. According to Slimani [15], the impact of a motor imagery-based training program on the activation levels of target muscles can have an effect on changes in neuronal control of muscles, through delimitation of the activity of the antagonistic muscles during exercise of the agonist muscle. The achievement of adequate levels of muscle strength and power is necessary for good performance outcomes in tennis. Consequently, Fontani [43] confirmed that a training program with motor imagery can strengthen physical performance based on the distal and proximal muscles of the lower and upper limbs. These mechanisms can explain the results observed in the present study in the middle (T2) and at the end of Ramadan (T3).

### 4.3. Limitations of the Study

The present study, despite its interesting results, has some shortcomings that should be properly acknowledged. For example, the evaluation of physical performance outcomes was carried out in the afternoon of the month of Ramadan, due to the modification of the training program imposed by this month, whereas the majority of tennis matches took place in the morning. This is another aspect that could probably influence performance outcomes because other studies have observed a decrease in physical performance in the afternoon but not in the morning [44]. Moreover, precise data on the hours of sleep of the participants were not recorded. Finally, the lack of data on the decisions taken by the receiver following the evaluation of the reaction time must be taken into account [25].

## 5. Conclusions

This study represents a first attempt to examine the effects of a motor imagery-based training program on physical performance outcomes in terms of agility, speed, and reaction time during Ramadan. Results showed that fasting during Ramadan reduced all performance outcomes in a sample of tennis players. A motor imagery training program after regular workouts may be an effective strategy to reduce the effect of fasting during Ramadan, by potentially stabilizing physical performance.

## Figures and Tables

**Table 1:** Descriptive statistics of the recruited sample.

Variables	Imagery Training Group	Control Group	*p*
	Mean (SD)	Mean (SD)	
Age (years)	16.9 ± 0.64	16.7 ± 0.59	0.41
Body mass (kg)	67.5 ± 2.96	66.12 ± 6.83	0.50
BMI (kg/m^2^)	22.02 ± 0.58	21.73 ± 0.87	0.31
Height (m)	1.75 ± 0.04	1.74 ± 0.06	0.70
Number of years of training	5.4 ± 1.2	5.7 ± 1.18	0.48
MIQ-RS			
VMI	5.2 ± 0.5	-	-
KMI	5.4 ± 0.4	-	-

Note. SD = standard deviation; BMI = body mass index; MIQ-RS = The Movement Imagery Questionnaire-Revised Second edition version; VMI = visual motor imagery; KMI = kinesthetic motor imagery.

**Table 2:** Averages (±SD) of daily calorie intake and percentages of carbohydrates, fats, and proteins recorded before and at the end of Ramadan.

Variables	Before Ramadan	End of Ramadan
	Mean (SD)	Mean (SD)
Calorie intake (kcal/day)	2956 ± 133.4	2947.0 ± 154.3
Protein (%)	18.7 ± 2.8	19.2 ± 2.2
Lipids (%)	31.2 ± 3.9	30.5 ± 4.1
Carbohydrate (%)	52.3 ± 5.1	52.1 ± 4.6

Note. SD = standard deviation.

**Table 3:** Averages (±SD) of body mass and body mass index (BMI) recorded before and during Ramadan.

Parameters	Groups	Before Ramadan (T0)	Beginning of Ramadan (T1)	Middle of Ramadan (T2)	End of Ramadan (T3)
		Mean (SD)	Mean (SD)	Mean (SD)	Mean (SD)
Body mass (kg)	Control group	66.12 ± 6.83	65.67 ± 6.88	65.35 ± 6.86 *	64.94 ± 6.89 *
	Imagery training group	67.52 ± 2.96	67.16 ± 2.85	66.82 ± 2.83 *	66.45 ± 2.87 *
Body mass index (kg/m^2^)	Control group	21.73 ± 0.87	21.58 ± 0.94	21.47 ± 0.93 *	21.33 ± 0.94 *
	Imagery training group	22.02 ± 0.58	21.91 ± 0.59	21.80 ± 0.57 *	21.68 ± 0.57 *

Note. SD = standard deviation; * Significantly different from before Ramadan at *p* < 0.001.
